# Time-dependent Enhanced Corrosion of Ti6Al4V in the Presence of H_2_O_2_ and Albumin

**DOI:** 10.1038/s41598-018-21332-x

**Published:** 2018-02-16

**Authors:** Yue Zhang, Owen Addison, Fei Yu, Brendy C. Rincon Troconis, John R. Scully, Alison J. Davenport

**Affiliations:** 1 School of Metallurgy & Materials, University of Birmingham, Birmingham, B15 2TT UK; 2 School of Dentistry, University of Birmingham, Birmingham, B5 7EG UK; 3 School of Dentistry, University of Alberta, Edmonton, T6G 1C9 Canada; 4 Medical College, Qingdao University, Qingdao, 266021 China; 5 Department of Mechanical Engineering, University of Texas at San Antonio, San Antonio, Texas 78249-0670 USA; 6 Centre for Electrochemical Science and Engineering, University of Virginia, Charlottesville, Virginia 22903 USA

## Abstract

There is increasing concern regarding the biological consequences of metal release from implants. However, the mechanisms underpinning implant surface degradation, especially in the absence of wear, are often poorly understood. Here the synergistic effect of albumin and H_2_O_2_ on corrosion of Ti6Al4V in physiological saline is studied with electrochemical methods. It is found that albumin induces a time-dependent dissolution of Ti6Al4V in the presence of H_2_O_2_ in physiology saline. Potentiostatic polarisation measurements show that albumin supresses dissolution in the presence of H_2_O_2_ at short times (<24 h) but over longer time periods (120 h) it significantly accelerates corrosion, which is attributed to albumin-catalysed dissolution of the corrosion product layer resulting in formation of a thinner oxide film. Dissolution of Ti6Al4V in the presence of albumin and H_2_O_2_ in physiological saline is also found to be dependent on potential: the titanium ion release rate is found to be higher (0.57 µg/cm^2^) at a lower potential (90 mV), where the oxide capacitance and resistance inferred from Electrochemical Impedance Spectroscopy also suggests a less resistant oxide film. The study highlights the importance of using more realistic solutions, and considering behaviour over longer time periods when testing corrosion resistance of metallic biomaterials.

## Introduction

Implantable medical devices are commonly fabricated from titanium (Ti) alloys. Ti alloys have suitable mechanical properties for many biomedical applications and possess excellent corrosion resistance which is dependent on the presence of a stable passive oxide film on its surface^1–3^. However, the peri-implant environment contains a variety of biological species which can act to modify corrosion behaviour, including proteins such as albumin and reactive oxygen species (ROS) such as hydrogen peroxide (H_2_O_2_)^4–6^. The effect of albumin alone, or H_2_O_2_ alone, on the corrosion of Ti alloys has been investigated^7–16^. However, the effect of combined exposure to both H_2_O_2_ and albumin on corrosion of Ti6Al4V has only recently been considered^17^. Yu *et al*. demonstrated that a combination of H_2_O_2_ and albumin can lead to significantly higher levels of corrosion of Ti6Al4V than either species alone^17^. In this paper, the mechanism proposed by Yu *et al*. to explain the synergistic action of albumin and H_2_O_2_ on Ti corrosion is explored, taking into account how the corrosion behaviour may change as a function of time.

Inflammation is ubiquitous with healing following implant insertion, but can also be present chronically in the peri-implant tissues associated with failing devices. Inflammation is associated with the production (by immune cells that have infiltrated the peri-implant tissues) of ROS species such as superoxide anions and H_2_O_2_^18,19^. Quantification of the levels of H_2_O_2_ in different environments in the body is complex and therefore there is a great variation in the way H_2_O_2_ exposures are simulated in *in-vitro* corrosion tests. ROS species have short half-lives and cellular production of ROS can take place over a wide range of time-scales, making quantification challenging^20^. However, it is accepted that peak concentrations of ROS can be reached when ROS release is confined in environmental niches, such as sub-cellular phagosomes or extracellularly between the cell and a material surface. H_2_O_2_ is known as a strong oxidant and can act to modify the corrosion resistance of Ti surfaces. Studies have been carried out to investigate the effect of H_2_O_2_ on corrosion of Ti alloys using concentrations ranging from 33 mM to 300 mM^7–10,17,21^. A number of studies have shown that H_2_O_2_ increases corrosion of Ti and its alloys by forming Ti(IV)-H_2_O_2_ complexes^6–9,17,21,22^, leading to formation of rougher, thicker and more porous surface oxide films.

Proteins are known to interact with metal oxides and modify the corrosion resistance of implants. Albumin is the most abundant protein (4.2–5.3%) in blood plasma and extracellular tissue fluid^4^. It is generally agreed that albumin inhibits cathodic reactions on Ti by adsorbing onto the metal surface and covering the reaction sites and/or blocking mass transport of dissolved O_2_^11,13,15–17^. However, it has also been claimed that albumin increases^11,12,17,23^, decreases^15,16,23^, or has no effect^14,24,25^ on anodic dissolution of Ti. Similar contradictory results have been found for other metallic biomaterials^26–28^. Metal-protein interactions are generally attributed to two effects: adsorption and chelation. In the case of adsorption, metal ion release is inhibited by a layer of adsorbed protein^15,16^. In the case of chelation, soluble metal-protein complexes are formed, enhancing the rate of dissolution^11,29,30^. Whilst albumin is the most prevalent protein in the tissue fluid, a wide range of proteins will interact with metal implant surfaces *in-vivo*. Different proteins can variably influence the corrosion susceptibility of the same metal surface^29,31^. For example, it has recently been shown that sequential exposure of albumin and fibrinogen to stainless steel modifies the corrosion rate^32^, and was attributed to displacement and exchange of the surface adsorbed albumin by the larger protein fibrinogen^32^.

Exposing Ti surfaces to H_2_O_2_ has been shown to modify subsequent interactions with adsorbed proteins, including albumin. Nagassa *et al*. showed that exposing Ti surfaces to H_2_O_2_ enhanced surface roughness and resulted in greater serum albumin adsorption^33^. Sousa *et al*. demonstrated differential binding affinities of albumin to a sputtered TiO_2_ surface when compared with a TiO_2_ surface that had been modified by H_2_O_2_ immersion^34^. Yu *et al*. found a synergistic effect of albumin and H_2_O_2_ on Ti6Al4V corrosion by measuring the rate of metal ion release after a 2 week immersion in physiological saline containing both species^17^. It was shown that albumin accelerated dissolution of Ti6Al4V in presence of H_2_O_2_ during the long term exposure. However, short term electrochemical tests showed contradictory findings where albumin appeared to supress the anodic reaction of Ti6Al4V in presence of H_2_O_2_. To explain this contradictory behaviour, the author suggested a mixed potential theory where an “active” dissolution region was proposed at low potentials and adsorption of albumin would supress the corrosion potential by taking it into this more “active” region of Ti^17^. However, the hypothesis needs to be validated carefully and more direct measurements reflecting temporal effects on dissolution rates need to be carried out.

The aim of this study is to understand the mechanism underpinning the synergistic effect of albumin and H_2_O_2_ on corrosion of Ti6Al4V as a function of time. Despite the various simulated physiological media for corrosion testing, 0.9% NaCl was chosen as a basic testing electrolyte (directed by ASTM F1875–98 and F1801-97) to control surface interactions that may further impact on interpretation of the interaction between albumin, H_2_O_2_ and the Ti6Al4V surface^35^. Both short term (<24 h) and long term exposures (up to 120 h) are investigated by electrochemical techniques in order to reflect possible changes in degradation of the metal surface, which is often projected as a slow and continuous process following the implantation in the body. Additionally, to test the “active” dissolution theory proposed by Yu *et al*.^17^, ion release was measured as a function of potential and parallel EIS measurements were carried at the static potentials to characterise the metal/oxide/solution interface.

## Results

### Corrosion under open circuit conditions

**Figure 1:**
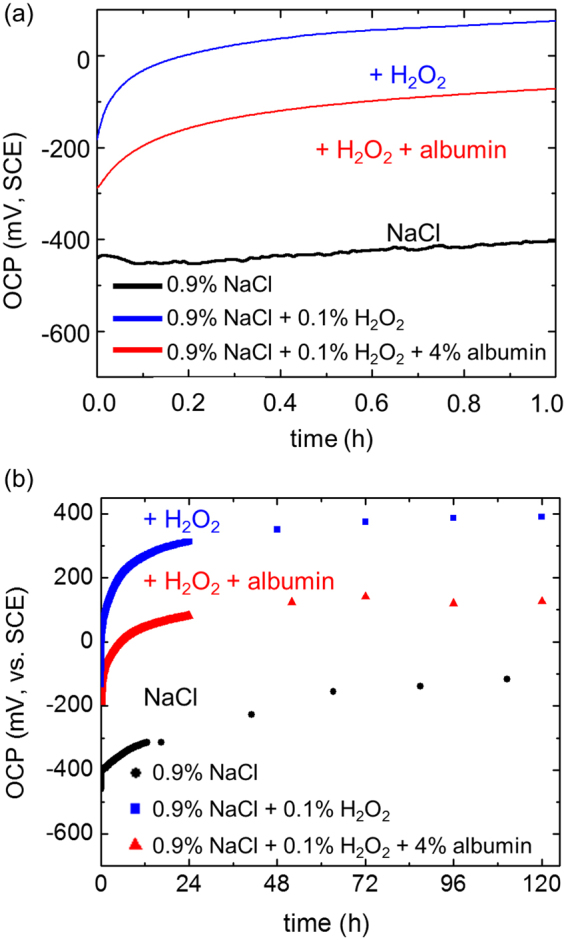
Open circuit potentials during exposure of Ti6Al4V in 0.9% NaCl with and without addition of 0.1% H_2_O_2_ or a combination of 0.1% H_2_O_2_ and 4% albumin at 37 °C; exposure for (**a**) the first hour; (**b**) 120 h.

Figure 1 shows the OCP (open circuit potential or corrosion potential) as a function of time in different solutions. In all cases, the corrosion potential gradually increased, consistent with a decrease in the anodic current density as a consequence of thickening of the oxide film and/or corrosion product layer on the metal surface. The OCP was consistently higher in a solution containing 0.1% H_2_O_2_ in 0.9% NaCl compared with that in 0.9% NaCl alone; this is likely to be due to an increased cathodic current since H_2_O_2_ is a strong oxidant^8,17^. In a solution containing 4% albumin, in addition to 0.1% H_2_O_2_ in 0.9% NaCl, the OCP was consistently lower than that in the presence of NaCl and H_2_O_2_ alone, but higher than in NaCl alone. This is consistent with previous work by Yu *et al*.^17^, showing that the presence of albumin can decrease the rate of the cathodic reduction of H_2_O_2_, probably by adsorption on the surface.

**Figure 2:**
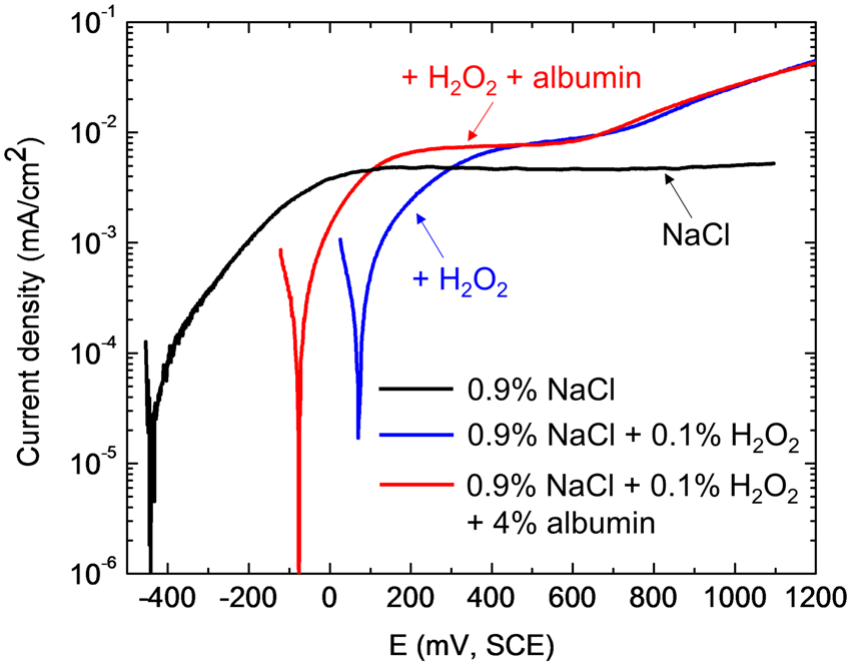
Potentiodynamic polarisation of Ti6Al4V after 1 h immersion at OCP in the solutions indicated at 37 °C. The scan rate was 1 mV/s.

Potentiodynamic polarisation curves can be used to confirm the interpretation of the OCP behaviour. Figure 2 shows anodic polarisation curves in the same set of solutions as those in Fig. 1. The order of the open circuit potentials is consistent with that in Fig. 1. The lowest OCP was observed in NaCl alone, with addition of H_2_O_2_ leading to a higher potential associated with a higher cathodic reaction rate (consistent with cathodic polarisation curves shown in reference^17^). Addition of H_2_O_2_ to NaCl also led to an increase in the anodic reaction rate above 400 mV (sufficiently above the OCP for the cathodic reaction to be negligible), with a small plateau from ~+400 to +600 mV followed by a steady increase. In the presence of NaCl, H_2_O_2_ and albumin, the anodic reaction above +400 mV was the same as that for NaCl and H_2_O_2_ alone, suggesting that albumin caused little or no change in the anodic reaction, but the cathodic reaction appears to be inhibited, explaining the relative values of the OCP shown in Fig. 1.

**Figure 3:**
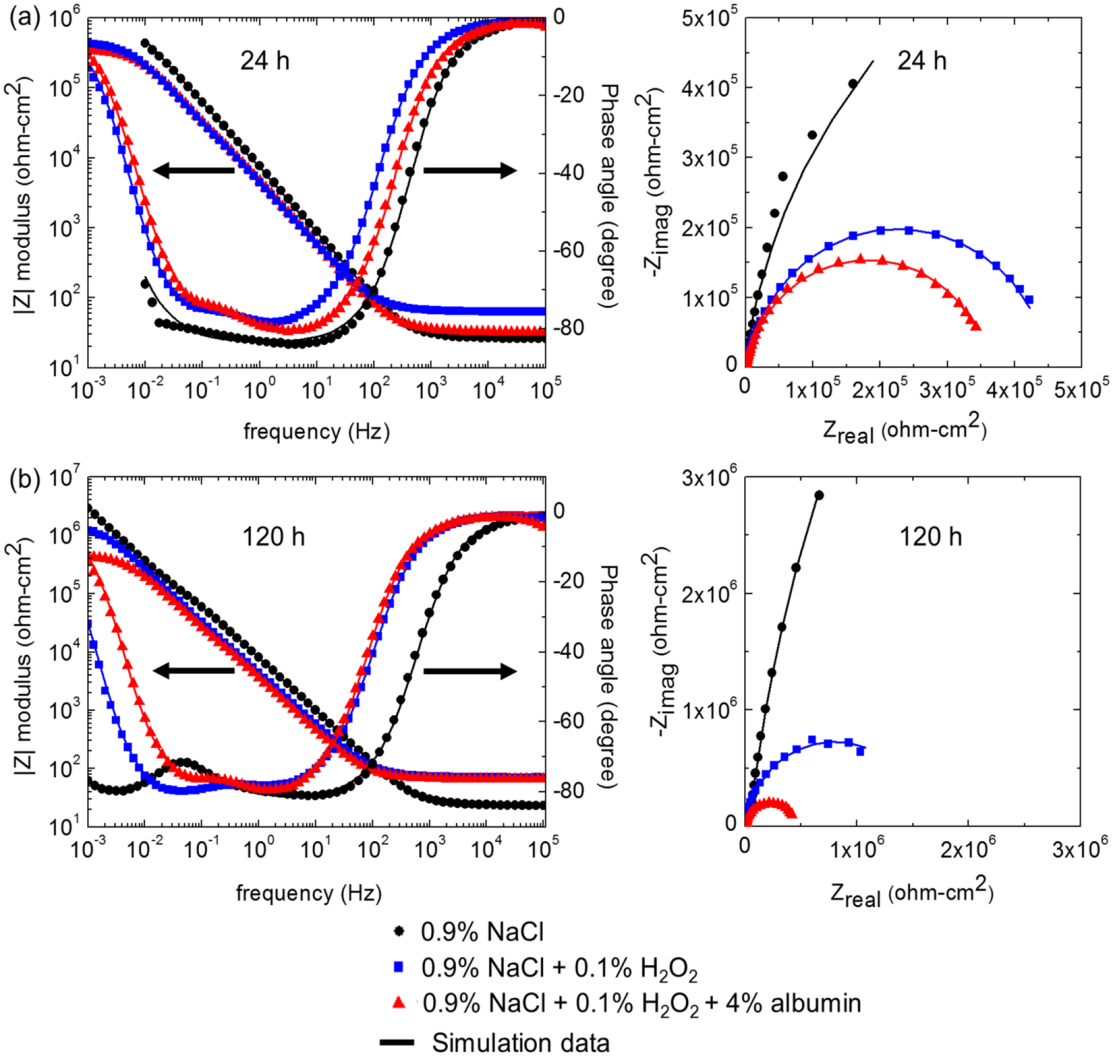
Bode and Nyquist plots of Ti6Al4V immersed at OCP in 0.9% NaCl in the absence and presence of 0.1% H_2_O_2_ and combination of 0.1% H_2_O_2_ and 4% albumin at 37 °C after (**a**) 24 h and (**b**) 120 h. Symbols are experimental data and lines are simulated data using the equivalent circuit shown in supplentary information Figure S1.

Figure 3 shows Bode and Nyquist plots of Ti6Al4V after immersion at OCP for 24 h and 120 h. Two time constants corresponding to the oxide film and likely the (double) layer were observed in the Bode plot after 24 h immersion, although this was less obvious for that in 0.9% NaCl than in the presence of 0.1% H_2_O_2_ and combination of 0.1% H_2_O_2_ and 4% albumin. Nevertheless, after 120 h immersion, more pronounced two time constants were presented in all solutions, which could also be associated with a two layer structured passive film. It can also be found that the real part of the impedance (diameter of semicircle in Nyquist plot, indicating the combined resistances of the passive film and the electrochemical reaction rates at OCP), increased with immersion time which is consistent with their OCP behaviour as observed in Fig. 1. It can also be found that resistance of Ti6Al4V in the presence of both albumin and H_2_O_2_ in 0.9% NaCl was lower than that the rest of the solutions at both measured time periods.

**Figure 4:**
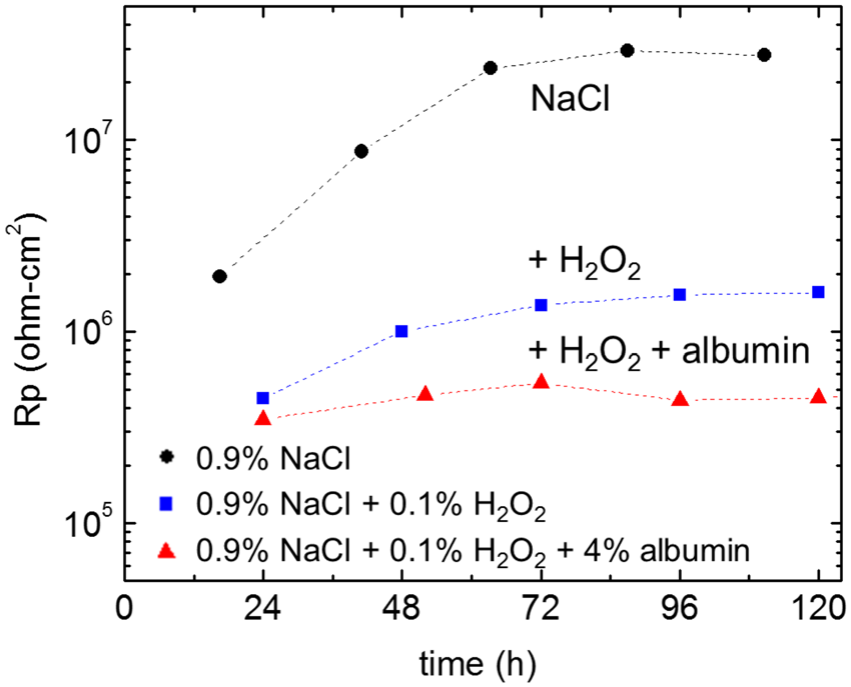
Polarisation resistance (R_p_) for Ti6Al4V as a function of immersion time in 0.9% NaCl with absence and presence of 0.1% H_2_O_2_ and combination of 0.1% H_2_O_2_ and 4% albumin at 37 °C. Values were calculated by fitting the data shown in Fig. 3 to the equivalent circuit (supplementary information Figure S1).

The data shown in Fig. 3 were fitted using the equivalent circuit shown in supplementary Figure S1. The polarisation resistance (R_p_) (Fig. 4) is the sum of two resistive components R_hf_ (oxide) and R_lf_ (inversely proportional to electrochemical reaction rate); it should be noted that R_p_ is dominated by R_hf_ (oxide resistance). Figure 4 shows that in all solutions, R_p_ gradually increased with immersion time and subsequently approached steady state at around 70 h. It can be seen that the presence of H_2_O_2_ reduced R_p_ by more than an order of magnitude after the initial measurement. At 24 h, the value of R_p_ in the presence of both albumin and H_2_O_2_ was similar to that in the presence of H_2_O_2_. However, at longer times, R_p_ in the presence of albumin and H_2_O_2_ was significantly lower than that in the presence of H_2_O_2_ alone.

**Figure 5:**
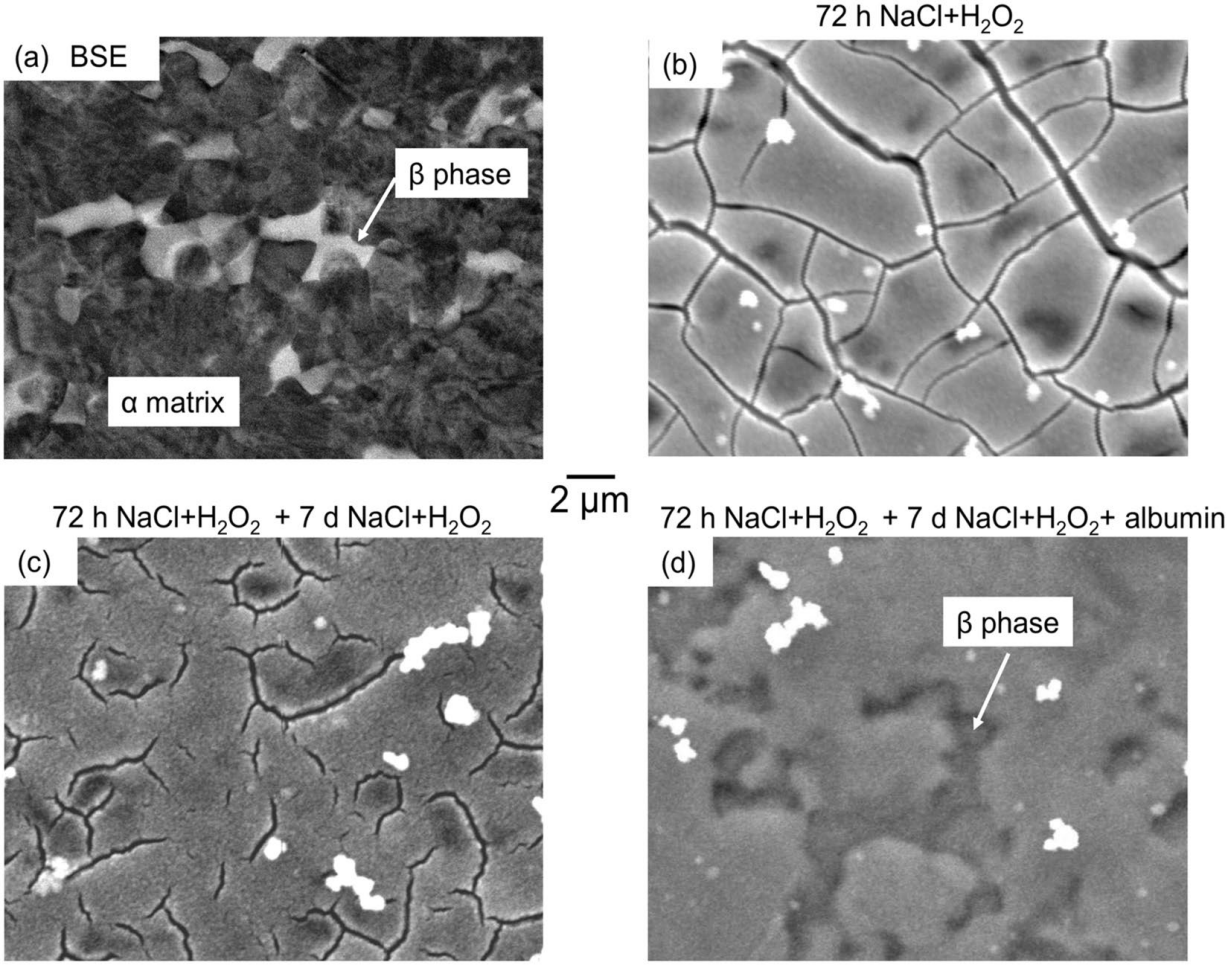
SEM images of the surface of Ti6Al4V (**a**) as-polished (backscattered electron micrograph, BSE) and secondary electron images after immersion in 0.9% NaCl + 10% H_2_O_2_ for 72 h at 37 °C (**b**); H_2_O_2_ pre-treated Ti6Al4V samples were then transferred to 0.9% NaCl + 0.1% H_2_O_2_ solution without (**c**) and with (**d**) presence of 4% albumin for 7 days at 37 °C. The presence of β-phase (identified with EDX, supplemntary Table S1) is indicated. The white precipitates presented in (**b**), (**c**) and (**d**) examined by EDX (see supplementary Table S1) were shown to be mostly Ti oxides.

Figure 5(a) shows the as-polished surface of Ti6Al4V with a distribution of β phase (identified with EDX, supplementary Table S1) in an α matrix. Figure 5(b) shows a secondary electron image of Ti6Al4V after immersion in 0.9% NaCl + 10% H_2_O_2_ for 3 days at 37 °C. The mud-crack morphology of corrosion products suggests the formation of a thick corrosion product layer. Figure 5(c) and (d) shows the morphology surfaces that were pre-treated in the same way as Figure (b) and then subsequently immersed 0.9% NaCl + 0.1% H_2_O_2_ without (c) or with (d) 4% albumin for 7 days at 37 °C. Figure 5(c) shows a similar mud-crack morphology to that in Fig. 5(b), but it can be seen that in the presence of albumin (Fig. 5(d)) the thick corrosion product layer was dissolved, and microstructural features such as β phase can be observed (detected with EDX, supplementary Table S1).

### Time-dependent corrosion behaviour at controlled potential

**Figure 6:**
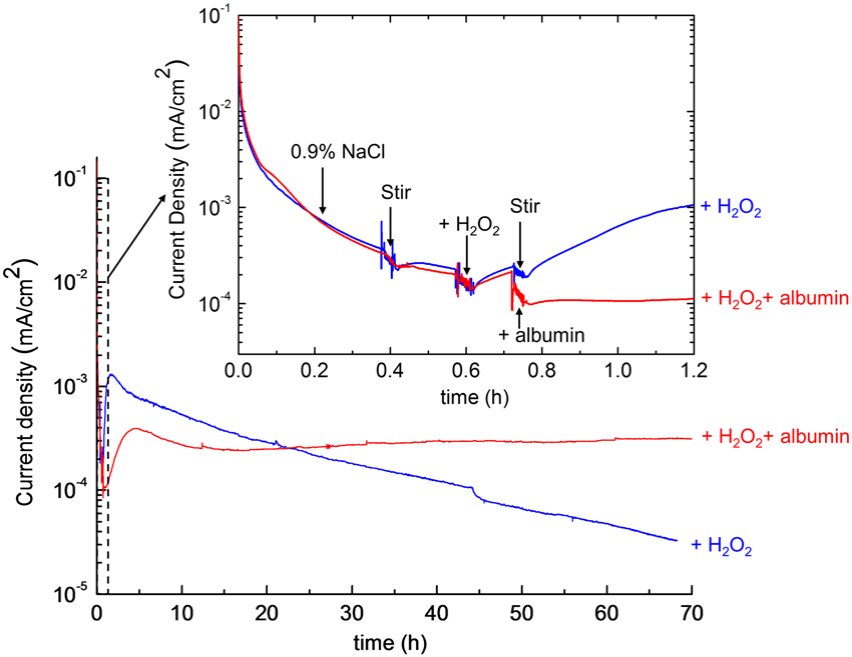
Current density as a function of time for Ti6Al4V at a fixed potential of 400 mV (vs. SCE) after 1 h immersion in 0.9% NaCl at OCP and 37 °C. The solution was stirred at 0.4 h, then followed by sequential additions of solutions to give a final concentration of 0.1% H_2_O_2_ and 4% albumin. Addition of solution was also made with the control group to give a final concentration of 0.1% H_2_O_2_, followed by stirring without addition of albumin. The early stage of the measurement is shown in the inset.

Figure 6 shows the current as a function of time for Ti6Al4V initially immersed in 0.9% NaCl at a constant potential of 400 mV with additions of 0.9% NaCl containing H_2_O_2_ or albumin. The potential was selected so that the effect on the anodic current density could be evaluated, but it should be noted that this value is a little higher than the interfacial potential under *in vivo* conditions. It can be seen that at an early stage (~1 h, see inset Figure), the current density increased after addition of solution to give a concentration of 0.1% H_2_O_2_ in 0.9% NaCl. For times up to 20 h, the solution with the additional 4% albumin had a lower current density compared with the control that contained only 0.1% H_2_O_2_ in 0.9% NaCl.

However, differences were revealed over longer exposure times. In the presence of H_2_O_2_ alone, the initial increase in current density was found to reach a maximum value of >1 µA/cm^2^ at ~1.7 h followed by a steady decay of almost two orders of magnitude, reaching ~0.03 µA/cm^2^ at 70 h. This is likely to be a result of the accumulation of corrosion products similar to those shown in Fig. 5(c). However, in the presence of both albumin and H_2_O_2_, the current density was found to be relatively constant after the initial increase without much of degradation. This is consistent with the observation of a much thinner corrosion product layer in Fig. 5(d), suggesting that albumin can promote dissolution of the H_2_O_2_-induced corrosion product, so the current reaches a steady state relatively quickly. A cross-over point in the current densities in these two solutions was observed at ~22 h and at 70 h, the current density in the presence of albumin and H_2_O_2_ was almost an order of magnitude greater than that in H_2_O_2_ alone.

### Metal ion release and EIS measurements at constant potential

Yu *et al*.^17^ proposed that the higher metal release in the presence of both H_2_O_2_ and albumin when compared with H_2_O_2_ alone, might be a consequence of albumin adsorption onto the metal surface, inhibiting the cathodic reaction and decreasing the potential to a proposed ‘active’ region for dissolution Ti6Al4V. To test this hypothesis, Ti6Al4V was exposed to 0.9% NaCl containing 0.1% H_2_O_2_ with and without 4% albumin for 24 h under potentiostatic control. Two potentials were chosen based on the OCP values observed in the two solutions after 24 h in Fig. 1(b). The OCP value in the presence of 0.1% H_2_O_2_ alone was close to 300 mV and in the presence of 0.1% H_2_O_2_ and 4% albumin a value closer to 90 mV was observed. Potential values of 90 mV and 200 mV were chosen so that they could be in the potential range of active/passive transition as proposed by Yu *et al*.^17^.

Table 1 shows that at both measured potentials, Ti ion release was significantly higher in the presence of albumin by almost a factor of 2, indicating that the effect of albumin in dissolving corrosion products dominates the synergistic effect of H_2_O_2_ and albumin in accelerating Ti ion release. However, in the presence of albumin and H_2_O_2_, Ti release was significantly higher at 90 mV than that at 200 mV (p < 0.001), supporting the hypothesis of Yu *et al*.^17^. In presence of H_2_O_2_ alone, slightly higher metal Ti ion release was observed at the lower potential, but the difference was much smaller (p < 0.01).

**Table 1:** The mean concentration and standard deviation of Ti ion release (µg/cm^2^) from Ti6Al4V after 1 h at OCP followed by 24 h at either 90 or 200 mV(SCE) in 0.9% NaCl with 0.1% H_2_O_2_ or 0.1% H_2_O_2_ + 4% albumin at 37 °C.

Ti ion release (µg/cm^2^)	90 mV	200 mV
0.9% NaCl + 0.1% H_2_O_2_	0.30 ± 0.01	0.28 ± 0.01
0.9% NaCl + 0.1% H_2_O_2_ + 4% albumin	0.57 ± 0.01	0.51 ± 0.01

Four measurements were made for each condition.

**Figure 7:**
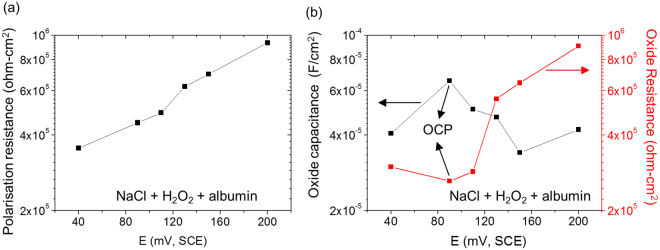
(**a**) Polarisation resistance and (**b**) Oxide resistance and capacitance of Ti6Al4V as a function of potential in 0.9% NaCl + 0.1% H_2_O_2_ + 4% albumin at 37 °C.

The metal/oxide/solution interface was also characterised by EIS measurements following potentiostatic polarisation for 20 h to develop stable passive film. Figure 7(a) shows the polarisation resistance as a function of potential, obtained by fitting the EIS spectra (supplementary Figure S2) with a two time constant equivalent circuit (supplementary Figure S1). The polarisation resistance increased steadily with potential from ~360 kΩ-cm^2^ at 40 mV to ~930 kΩ-cm^2^ at 200 mV. Figure 7(b) shows the oxide resistance (R_hf_) and capacitance (calculated from Y_hf_ using equation (2)) as a function of potential. The oxide resistance showed little change from 40 to 90 mV then increased significantly with potential up to 200 mV. The oxide capacitance showed some variation with potential, but appeared to give a maximum value at 90 mV.

## Discussion

It can be seen from potentiodynamic polarisation (Fig. 2) that the addition of H_2_O_2_ increased the passive current density of Ti6Al4V in 0.9% NaCl. Similarly, the potentiostatic current density (Fig. 6) increased after addition of H_2_O_2_ in 0.9% NaCl. H_2_O_2_ is known to increase the corrosion rate of Ti^22^, and its alloys^7^, via a complexation reaction with Ti ions^6,8,9,21^.

At short time exposures (t < ~22 h), addition of 4% albumin suppressed the current density in the presence of H_2_O_2_ (Fig. 6). Rapid adsorption of albumin onto the Ti6Al4V oxide surfaces has previously been observed with Quartz Crystal Microbalance (QCM) measurements and X-ray photoelectron spectroscopy (XPS)^34,36^. In addition, using ^125^I radio labelling, it has been shown that albumin adsorbs on oxidised Ti surfaces from the early stages of immersion with most of the adsorption taking place within the first few minutes^37^. To date, most studies have reported that albumin inhibits corrosion of commercially pure (CP) Ti and Ti alloys^15,16,23^, and also protects it from dissolution induced by e.g. fluoride ions^38^. Adsorption of albumin films have been proposed to block dissolution. However, all of these experiments have taken place over short time periods, typically over no more than a few hours.

After longer term exposures (up to 72 h), it can be seen from Fig. 6 that the potentiostatic current density in the presence of H_2_O_2_ alone decreased significantly after the initial increase, most likely as a result of blocking of the surface by the peroxide corrosion product layer shown in Fig. 5(b,c). It has been proposed that the Ti oxide film forms a two-layer structure in phosphate buffered saline, which consists of a thin barrier type inner layer and thick porous type outer layer, and the presence of H_2_O_2_ reduces corrosion resistance and thickens the outer porous layer^8,9,21^.

In contrast, prolonged exposure in the presence of both H_2_O_2_ and albumin resulted in a higher current density than that in the presence H_2_O_2_ alone, with a cross-over point at ~22 h. This is consistent with the observation of a much thinner corrosion product layer in Fig. 5(d), suggesting that albumin promotes dissolution of the peroxide corrosion product layer at longer time periods.

The interaction between albumin and peroxide corrosion layer has been studied by Sousa *et al*. via a kinetic perspective of albumin adsorption, adhesion and exchangeability (desorption)^34^. It was found that the more hydrophilic surface of TiO_2_ after H_2_O_2_ immersion adsorbed less albumin but with higher work of adhesion i.e. it was more strongly attached on the surface, compared with that of the more hydrophobic surface of sputtered TiO_2_^34^. After the adsorption step, it was suggested that the exchangeability of albumin was changing with time: after 24 h the adsorbed albumin molecules on the H_2_O_2_-treated TiO_2_ surface seem to be less exchangeable than those adsorbed on the sputtered TiO_2_ surface; however after 72 h nearly all the adsorbed albumin molecules effectively exchanged with other albumin molecules, which suggested that longer time is needed for exchange of albumin molecules adsorbed on surface of H_2_O_2_-TiO_2_ complexes^34^. Therefore, the time dependent dissolution behaviour observed in the presence work is consistent with the desorption of albumin-metal complexes as it is a slow, rate determining process^34^, and can result in thinner oxides.

The time-dependent data were confirmed by the EIS data shown in Fig. 4. The polarisation resistance (R_p_) of Ti6Al4V in in the presence of H_2_O_2_, R_p_ was significantly lower than that in NaCl alone after 24 h and both increased gradually with time, consistent with the formation of a thicker corrosion product layer. At 120 h, R_p_ in presence of H_2_O_2_ was over an order of magnitude lower than that in NaCl alone. Comparable values were measured for CP Ti in PBS where R_p_ decreased from 2 × 10^7^ ohm-cm^2^ to 8 × 10^5^ ohm-cm^2^ in the presence of 50 mM H_2_O_2_ (compared with 33 mM in the present work) after 125 h^39^. The decreased corrosion rate of Ti6Al4V in the presence of H_2_O_2_ over time has been attributed to growth of a corrosion product layer.

In the presence of albumin and H_2_O_2_, R_p_ showed little change over time compared with the behaviour in H_2_O_2_ alone, and it can be seen from Fig. 4 that after 120 h, R_p_ in presence of H_2_O_2_ and albumin was significantly lower than that of H_2_O_2_ alone, consistent with the presence of a thinner corrosion product layer and increased dissolution. This observation correlates well with previous ICP-MS measurements carried out by Yu *et al*.^17^, where metal ion release was measured in identical solutions after 2-week incubation and the combination of H_2_O_2_ and albumin was found to give much higher Ti release than that of H_2_O_2_ alone. It suggests that the explanation for this observation is likely to be the increased dissolution of the peroxide corrosion product in the presence of albumin.

In the work of Yu *et al*., short term electrochemistry tests (t ~ 1 h) and long term (2 weeks) ion release measurements showed contradictory results regarding the effect of albumin on dissolution of Ti6Al4V in the presence of H_2_O_2_ in physiological saline. The investigation explained the observation using a proposed mixed potential theory, suggesting that albumin suppresses the cathodic reaction, decreasing the corrosion potential into a more “active” region for dissolution of Ti at lower potential^17^. In the present work, this hypothesis was tested by measuring metal ion release from Ti6Al4V at constant applied potentials. Table 1 shows that in the presence of albumin and H_2_O_2_, the rate of Ti ion release was higher at lower potentials, consistent with the proposed “active dissolution” effect^17^. However, it is clear that the increased metal ion release at both potentials is substantially greater in the presence of albumin, indicating that the synergistic effect of albumin and peroxide observed at longer exposure times is dominated by the role of albumin in dissolving the peroxide corrosion product.

Metal implants are exposed to an environment that is more complex than the physiological saline (0.9% NaCl) that is used in standard tests such as ASTM F1801-97 and ASTM F1875-98^40,41^. In the present work on Ti6Al4V, the major protein in the body, albumin, is shown to interact with the corrosion products of Ti in the presence of H_2_O_2_, which is produced in inflammatory processes. The level of H_2_O_2_ used in this work (0.1% = 33 mM) is at the lower end of those used in previous *in vitro* corrosion studies^7–10,17,21^. Concentrations of H_2_O_2_ measured in extracellular environments are frequently reported to be in the µM range^18–20,42,43^. However, both immune cells and microbial biofilms (such as those found on oral implants) create local micro-environments where H_2_O_2_ can be considerably concentrated reaching mM concentrations. For example, measurements of H_2_O_2_ concentration in surface biofilms of the oral microbe *Streptococcus gordinni* have been shown to increase with proximity to the biofilm with quasi-steady-state concentrations of 1.4 mM measured at 100 µm from the exposed microbial surface^43^. It was proposed that the concentration of H_2_O_2_ may reach much higher levels within the biofilm itself and at the biofilm substrate interface.

More recently, the effect inflammatory cell induced corrosion has been recognised as an important mechanism for *in vivo* metal degradation^42,44,45^. It has been found that during inflammation, the cellular physiology produces a localized acidic^45^ and concentrated ROS environment^42^, which can lead to accelerated corrosion of implants and generation of metallic particulates and ions. These implant derived products have themselves been shown to elicit innate and adaptive cellular responses including release of pro-inflammatory cytokines^46^, leading in some cases to persistent peri-implant inflammation, bone resorption and aseptic loosening of the device^46–48^.

This work also highlights the need to examine the time-dependence of corrosion behaviour of metals used for biomedical implants, since in this case there is a clear switch in the observed behaviour at ~1 day. Similar time-dependent corrosion behaviour in the presence of albumin was also found in biodegradable Mg alloys in simulated body fluid^49^. It was found that albumin blocks dissolution in the first few hours of exposure but increased corrosion was observed over longer exposures^49^. As biomolecules such as albumin and H_2_O_2_ are present in inflamed peri-implant tissue, these observations raise the question of whether standard testing should be complemented by testing in more realistic environments, over more realistic time periods, and using enhanced corrosion characterisation methods rather than standard polarisation curves^50^.

## Methods

### Materials and surface preparation

Ti6Al4V (ASTM Grade 5) was commercially sourced in disc shape specimens of 14 mm diameter and 1.2 mm thickness (Titanium Products Ltd, UK). The composition (max. wt%) was 0.05N, 0.08C, 0.015H, 0.3 Fe, 0.2O, 3.5–4.5V, 5.5–6.8 Al, with the balance Ti. Samples for electrochemical tests were cold-mounted in a non-conducting setting resin. To ensure a reproducible surface finish, all samples were wet polished from 800 to 4000 grit using SiC abrasive paper and finished with an OP-S colloidal silica suspension (0.04 μm) on MD-Chem cloth (Struers, Ballerup, Denmark). Samples were cleaned thoroughly with deionised water (Millipore, >15 MΩcm), rinsed with 100% methanol and finally dried in cool air stream. To ensure consistency in the surface oxide layer between different samples, each sample was exposed in ambient lab air for a fixed time of 15 min following surface preparation prior to electrochemical experiments.

### Test solutions and electrochemical cells

Physiological saline (0.9% NaCl, 0.15 M) solutions were prepared by dissolving NaCl (Sigma Aldrich, UK) in deionised water. H_2_O_2_ (30 wt.% in H_2_O, Sigma Aldrich, UK) was added to 0.9% NaCl to give a concentration of 0.1% (33 mM). Bovine serum albumin (≥98%, lyophilized powder, Sigma Aldrich) was dissolved in 0.9% NaCl containing 0.1% H_2_O_2_ at concentration of 4%. Both reagent chemicals i.e. 30 wt% H_2_O_2_ and bovine serum albumin powder (${\rm{\ge }}$98%) were stored at 4 ± 2 °C and were used to prepare fresh solutions for each electrochemical experiment. A double-wall three-electrode cell was used for both DC and AC electrochemical tests. The cold mounted Ti6Al4V samples were used as working electrode, Pt mesh as counter electrode and saturated calomel electrodes (SCE) as a reference electrode. All potentials are referred to this scale in this paper. The cell was maintained at a temperature of 37 ± 1 °C by a circulating heated water jacket.

### DC electrochemical tests

A Gill AC potentiostat (ACM instruments) was used for DC electrochemical tests. Samples were immersed in a test solution at the OCP for 1 h, followed by potentiodynamic polarisation from −50 mV (vs. OCP) at a scan rate of 1 mV/s. Each test was repeated three times to confirm consistency. In addition to potentiodynamic tests, potentiostatic polarisation at 400 mV was conducted after 1 h immersion at OCP in 0.9% NaCl, followed by sequential additions of 0.1% H_2_O_2_ with and without 4% albumin. Solutions were stirred during addition of species to ensure complete mixing. Solution of 0.9% NaCl containing H_2_O_2_ was added at ~0.6 h to give final concentration of 0.1% H_2_O_2_ and solution of 0.9% NaCl containing albumin was added at ~0.74 h to give final concentration of 4% albumin. The control solution without any addition of species was stirred at the same times.

### Electrochemical Impedance Spectroscopy (EIS)

A Gamry PCI4 potentiostat (Gamry instruments) was used to measure EIS. EIS measurements were performed at open circuit potential perturbing with 10 mV RMC AC signal with a wide frequency range from 10^−3^ to 10^5^ Hz. The acquisition rate was 10 points per decade. Measurements were taken after immersion in the solution for 24 h intervals up to 120 h. Additional samples were held at constant potentials for 20 h after which EIS was measured at the constant potential. A two time constant electrical circuit (supplementary Figure S1) was used to fit EIS data, which consists of two parallel Constant Phase Element (CPE) and resistor pairs in series with solution resistance (R_s_)^51,52^. Fitting of EIS data was performed by Gamry Echem Analyst software with simplex algorithm method and the quality of fitting was judged by chi squared value (χ^2^) < 1 × 10^−3^. Quantitative parameters were obtained. Polarisation resistance was calculated from the sum R_hf_ and R_lf_, corresponding to resistances of inner compact and outer porous passive layer respectively. The impedance of a CPE is given by

1 $$Z(CPE)={{Y}_{0}}^{-1}{(j\omega )}^{-n}$$

The capacitance was converted from the CPE using the Mansfield equation^53^:

2 $$C={Y}_{0}{({{\omega }^{^{\prime\prime} }}_{m})}^{a-1}$$

where ${{\omega }^{^{\prime\prime} }}_{m}$ is the angular frequency at which the imaginary part of impedance has a maximum^53^.

### Ion release measurements

Samples were potentiostatically polarised at 90 or 200 mV for 24 h after 1 h immersion at OCP in 0.9% NaCl with presence of 0.1% H_2_O_2_ or combination of 0.1% H_2_O_2_ and 4% albumin. Solutions were then extracted and released ions of interests (i.e. Ti, Al, V) were analysed by Butterworth Laboratories (UK) using inductive coupled plasma mass spectroscopy (ICP-MS) (Agilent 8800 ICPMS triple quad). The working quantitation limit for Ti is 10 µg/L, Al is 100 µg/L and V is 10 µg/L. 5 mL sample aliquots were measured and diluted up to 1000 mL with water and diluent containing 1.5 mL of Triton-x-100, 0.15 mL of 20% ammonia and 15 mL of 1.4 mM EDTA. The ion release results for each solution were compared across the potentials through one-way analysis of variance (ANOVA) at a significance level of α = 0.05. A total number of four repeats were performed for each condition.

### Incubation test and SEM surface analysis

Ti6Al4V discs were mirror polished on both sides and ultrasonically cleaned with deionised water and methanol for 10 min respectively. Surface prepared discs were incubated in saline with 10% H_2_O_2_ solution for 3 days in thermostatic chamber at 37 ± 1 °C. The H_2_O_2_ treated samples were then transferred to 0.1% H_2_O_2_ saline solutions with and without addition of 4% albumin for 7 days. Samples before and after incubation were examined by SEM JEOL 6060 at an acceleration voltage of 20 kV in both secondary electron (SE) and back scattered electron (BSE) mode.

### Data availability

The datasets generated and analysed during the current study are available from the corresponding author on reasonable request.

## Conclusions

Albumin was found to have a time dependent effect on dissolution of Ti6Al4V in the presence of H_2_O_2_ in 0.15 M NaCl.

At short times (t < ~22 h), albumin supressed dissolution rates, but at longer times, the rate of dissolution increased to values greater than those found in absence of albumin.

The long term dissolution behaviour in the presence of albumin and H_2_O_2_ can be attributed to the enhanced dissolution of peroxide corrosion products in the presence of albumin, resulting in formation of a thinner oxide layer on the metal surface.

The rate of metal ion release in the presence of albumin and H_2_O_2_ was found to depend on potential. At lower potential, a higher metal ion release rate was observed via ICP-MS measurements, and the oxide resistance and capacitance determined from EIS was consistent with a thinner oxide layer than that found at higher potentials.

The corrosion rate of Ti6Al4V in the presence of albumin and H_2_O_2_ in 0.15 M NaCl was observed to be significantly higher than that in 0.15 M NaCl alone, emphasising the need to test alloys for biomedical implants in more realistic physiological solutions. It is also essential to make measurements over longer time periods than those typically performed in standard tests involving conventional polarisation curves.

## Electronic supplementary material

Supplementary Information
