# Supplementary material for: Time-dependent Enhanced Corrosion of Ti6Al4V in the Presence of H2O2 and Albumin
Source: Sci Rep. 2018 Feb 16;8:3185. doi: 10.1038/s41598-018-21332-x (PMC5816596; doi:10.1038/s41598-018-21332-x)
Supplement: Supplementary file 1 — Supplementary Information [file 41598_2018_21332_MOESM1_ESM.pdf]

Supplementary Information

Time-dependent Enhanced Corrosion of Ti6Al4V in the Presence of H_2O_2 and Albumin

Yue Zhang[1], Owen Addison[2,*], Fei Yu[1,3], Brendy C. Rincon Troconis[4], John R. Scully[5], Alison J. Davenport[1]

[1]School of Metallurgy & Materials, University of Birmingham, Birmingham, B15 2TT, UK

[2]School of Dentistry, University of Birmingham, Birmingham, B5 7EG, UK

[3]Medical College, Qingdao University, Qingdao 266021, China

[4]Department of Mechanical Engineering, University of Texas at San Antonio, San Antonio, Texas 78249-0670, USA

[5]Centre for Electrochemical Science and Engineering, University of Virginia, Charlottesville, Virginia 22903, USA

*corresponding author

Email addresses: yxz159@bham.ac.uk (Y. Zhang), o.addison@bham.ac.uk (O. Addison), feiyu@qdu.edu.cn (F. Yu), brendy.rincon@utsa.edu (B. C. Rincon Troconis), jrs8d@virginia.edu (J. R. Scully), a.davenport@bham.ac.uk (A. J. Davenport)

Table S1. A summary of the mean compositions (n=3) with a range of compositions from regions of interests by EDX analysis of the as-polished Ti6Al4V surface (Figure 5a) and the H_2O_2 pre-treated surface following incubation in an albumin containing solution (Figure 5d).

Elemental% (wt%)		Ti	Al	V	O	Na
as-polished surface (Figure 5a)	α phase	91.2 (90.7-91.7)	6.1 (5.7-6.6)	2.5 (2.3-2.8)	0	0
	β phase	86.3 (84.8-88.6)	4.4 (3.9-4.9)	10.0 (8.2-11.3)	0	0
surface after immersion in an albumin containing solution (Figure 5d)	white precipitates	71.2 (70.4-72.1)	3.0 (2.9-3.1)	2.1 (1.7-2.6)	22.4 (21.8-23.2)	1.4 (1.3-1.6)
	Oxides (α phase)	81.0 (80.4-82.0)	3.0 (3.0-3.1)	2.9 (2.4-3.7)	12.1 (11.7-12.6)	0
	Oxides (β phase)	82.1 (81.1-82.9)	2.7 (2.4-3.2)	3.0 (2.5-3.5)	11.5 (10.4-12.3)	0

Figure S1

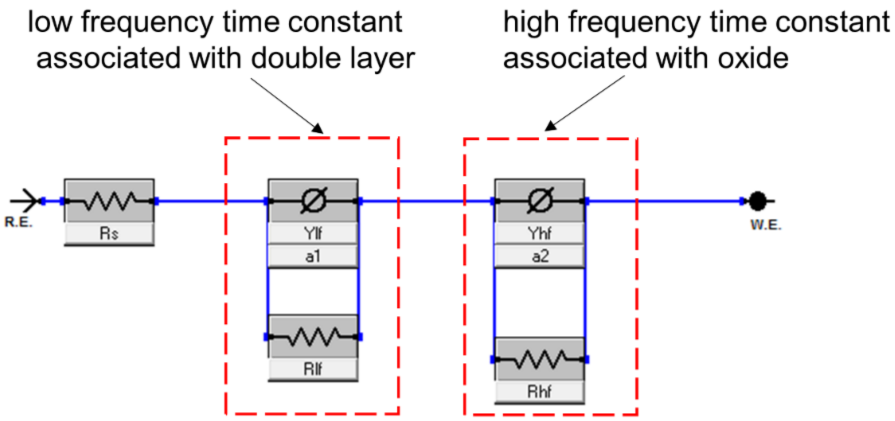

Figure S1. Equivalent two time constants circuit in generation of simulation data.

Figure S2

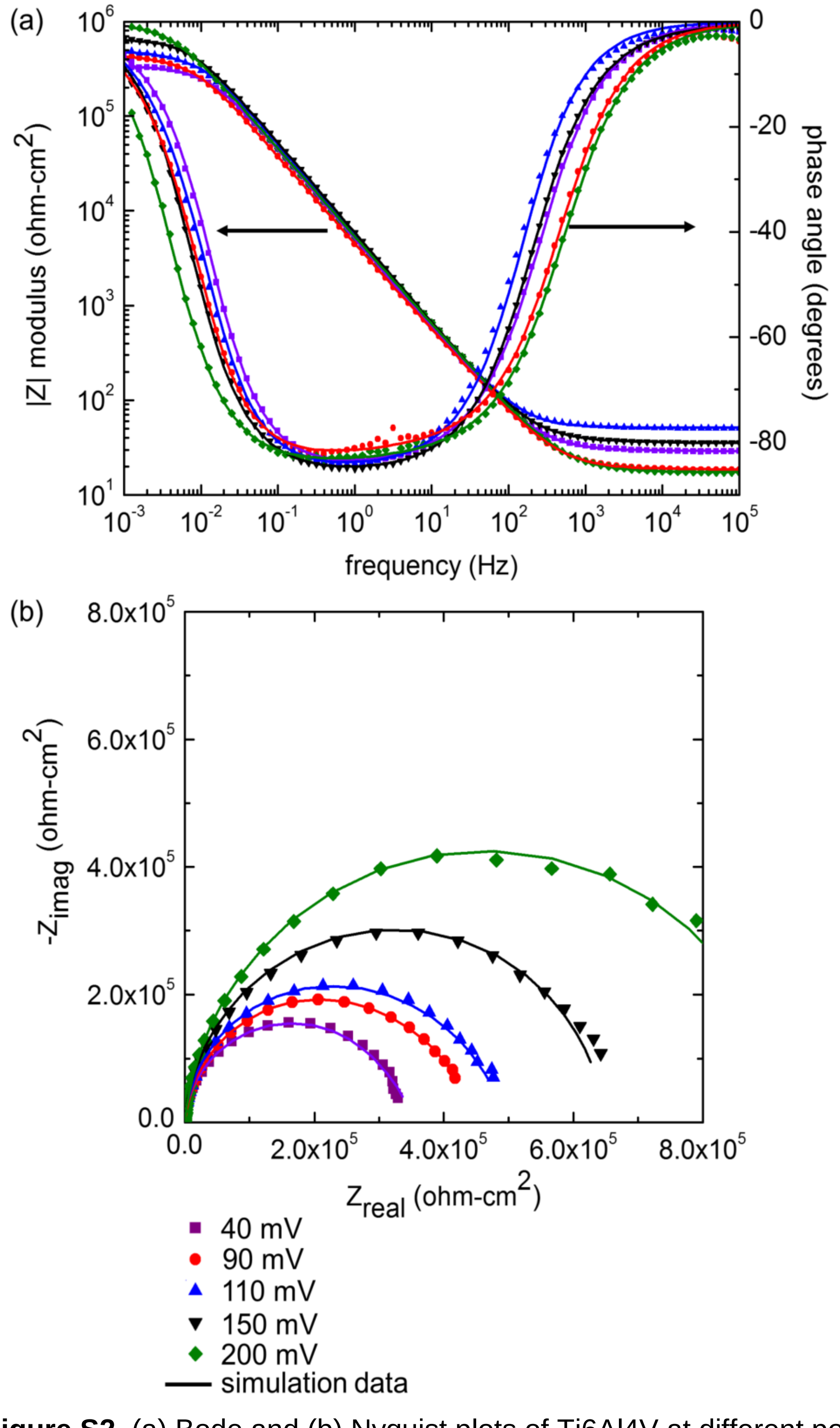

Figure S2. (a) Bode and (b) Nyquist plots of Ti6Al4V at different polarised potentials which were held statically for 20 h after 1 h immersion at OCP in 0.9% NaCl + 0.1% H_2O_2 + 4% albumin at 37 °C.
